# Application of Moringa (*Moringa oleifera*) as Natural Feed Supplement in Poultry Diets

**DOI:** 10.3390/ani9070431

**Published:** 2019-07-09

**Authors:** Shad Mahfuz, Xiang Shu Piao

**Affiliations:** State Key laboratory of Animal Nutrition, College of Animal Science and Technology, China Agricultural University, Beijing 100193, China

**Keywords:** *Moringa oleifera*, poultry, growth performance, laying performance, health status

## Abstract

**Simple Summary:**

The wide application of in-feed antibiotics in poultry production has created public health hazards. A driving force for the interest of using natural herbs is to establish the antibiotics alternative in poultry production that has been reported in the literature. Therefore, the objective of the current review is to determine the effects of moringa (*Moringa oleifera*) tree leaves, seeds and their extracts on chickens’ performance and health status. Based on previous findings, *M. oleifera* as natural feed supplement has sustained the production performance and improved the health status in chickens.

**Abstract:**

Application of natural herbs with a view to enhancing production performance and health status has created an important demand in poultry production. With the increasing concerns on this issue, greater attention paid to alternatives to antibiotics for organic meat and egg production has led to a great demand. This study was conducted with view to assessing the possible role of *M. oleifera* as a natural feed supplement in poultry ration. Various scientific findings and published research articles were considered concerning issues including the study background, objectives, major findings, and conclusions of the review. *M oleifera* is known as a miracle tree because of its wealthy resource of various nutrients with high biological values. *M. oleifera* has been used as a growth promoter, immune enhancer, antioxidant, and has a hypo-cholesterol effect on chickens. It has both nutritional and therapeutic values. However, there is still much confusion in past published articles involving the major roles of *M. oleifera* in production performance and health status of chickens. Taking this into account, the present study highlights an outline of the experimental uses of *M. oleifera* on growth performance, egg production performance, egg quality, and health status in broilers and laying hens justified with the past findings to the present. The knowledge gaps from the past studies are considered, and the feasibility of *M. oleifera* in poultry ration is suggested. The findings have motivated further study on *M. oleifera* to find out the most active ingredients and their optimal doses in both broiler and laying hen rations. Finally, the present study highlights that supplementation of *M. oleifera* may play a role in the immunity, sound health, and production performance in poultry.

## 1. Introduction

The human population is increasing globally day by day. Meeting the increasing demand of animal protein and providing safe food for human beings that is free from antibiotics by using herbal feed resources is a great challenge for the animal scientists in the future. The issue considering antibiotic resistance has created an augmented force to reduce antibiotic uses in livestock and poultry production [1,2]. Dietary inclusion of herbs and their extracts has growth-promoting roles in poultry [3]. Furthermore, different natural medicinal plants and their extracts as feed supplements have been used as a substitute for antibiotics in poultry production [4,5]. In addition, Mahfuz et al. [6] reported that poultry scientists are now dedicated to applying unconventional natural feed supplement, which may play a role in possible therapies to improve the health as well as production performance of chickens.Thus, poultry researchers are searching for potential natural feed resources that will be both environmentally friendly and safe for human society [7,8]. 

*Moringa oleifera* is a well-known cultivated species in the genus *Moringa*, (family Moringaceae) under the order Brassicales. The common names of *Moringa oleifera* include moringa, drumstick tree, horseradish tree, and ben oil tree or benzoil tree or miracle tree [9,10,11]. The *M. oleifera* tree is native to South Asia, especially India, Sri Lanka, Pakistan, Bangladesh, Afghanistan; North Eastern and South Western Africa, Madagascar, and Arabia [12,13,14,15]. The moringa seed and leaves have a broad use in the food industry and therapeutic issues [12]. It is popular for its seeds, flowers and leaves inhuman food and as herbal medicine [16]. The different parts of the *M. oleifera* tree are used as a good source of human nutrition and in traditional diets in different countries of the world [17,18]. Furthermore, the seed powder of *M. oleifera* contains polyelectrolytes, which are the most important active ingredients for water purification [18,19]. 

*Moringa oleifera* is very useful as a feed supplement for animals, as its leaves are highly nutritious. The leaves of *M. oleifera* are the most nutritious part, being a significant source of vitaminB complex, vitamin C, pro-vitamin A as beta-carotene, vitamin K, manganese, and protein among other essential nutrients [20]. *Moringa oleifera* leaves have antimicrobial roles and are rich with fats, proteins, vitamins, and minerals [18,21]. The extracts from leaves of *Moringa oleifera* contain low amounts of polyphenols, which might have effects on blood lipid metabolism [20,22]. *Moringa oleifera* can be used as a source of micronutrient and as a dietary supplement in poultry [23,24]. In addition, *Moringa oleifera* leaf powder has anti-septic and detergent properties due to presence of different phytochemicalsin the leaves [25]. *Moringa oleifera* was reported to be an excellent source of vitamins and amino acids that reportedly boost immune systems [17]. The seed extracts of moringa are rich in polyunsaturated fatty acid [26,27]. *Moringa oleifera* exhibits anti-oxidant properties that can suppress formation of reactive oxygen species (ROS) and free radicals [27,28]. 

Until the present day, the application of *M. oleifera* in farm animals to improve the production performance and health status has been limited. Even though it was established that *M. oleifera* has medicinal importance for the health of chickens, unfortunately the inclusion levels of *M. oleifera* in poultry ration and their mode of actions are still under consideration. Taking this into consideration, the present study focuses on uses of *M. oleifera* as a natural feed supplement as well as an alternative to antibiotics that can improve the performance and health status of chickens.

## 2. Biological Role of *M. oleifera*

**Figure 1:**
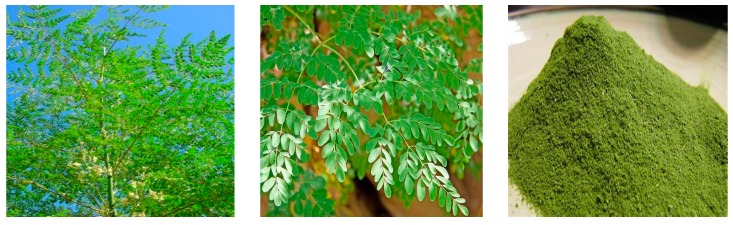
*Moringa oleifera* tree, tree leaves, and leaves powder.

The *M. oleifera* tree is globally known for its economic and therapeutic roles (Figure 1). Ithas been honored as the “Botanical of the Year 2007” by the National Institute of Health (USA), [11]. The tree is also known as “never die” or “miracle tree”to the people of Africa [11]. Now the application of *M. oleifera* leaves in preparing foods is receiving great attention. Peoples from Ghana, Nigeria, Ethiopia, East Africa, and Malawi are consuming the moringa tree leaves directly in their diets [29]. Furthermore, *M. oleifera* leaves have been used for making soups, foods, breads, cakes, and yoghurts [30,31,32,33].

### 2.1. Antioxidant Properties of M.oleifera

*M. oleifera* tree leaves possess various phytochemicals that have antioxidant properties and roles in controlling a wide range of diseases, like diarrhea, asthma, and various cancers [11]. The leaves of *M. oleifera* have also been reported to hold extensive amounts of total phenols, proteins, calcium, potassium, magnesium, iron, manganese, and copper [33]. They also contain rich sources of different phytonutrients, such as carotenoids, tocopherols, and ascorbic acid, which are good sources of dietary antioxidants [34,35]. A significant increase in activities of superoxide dismutase (SOD), catalase (CAT), glutathione-S-transferase (GST), and a decrease in lipid peroxide (LPS) content were found in moringa leaf extracts [11]. In addition, leaves extract from *M. oleifera* could improve the superoxide dismutase (SOD), catalase, glutathione, and peroxidase levels and reduce lipid peroxidation in albino mice [36]. Furthermore, total phenolic, flavonoid, and flavonol content in leaf extracts was found to be 120 mg/g of gallic acid equivalents (GAE), 40 mg/g of GAE, and 12.12 mg/g of GAE, respectively [37,38].

### 2.2. Therapeutic and Antimicrobial Properties of M.oleifera

*M. oleifera* leaf extracts have been distinguished as having anticancer, cytotoxic, anti-proliferative, anti-leukemia, anti-hepatocarcinoma, and chemo-protective properties [39,40,41]. The antitumor function of leaf extracts of *M. oleifera* is associated with the antioxidant and apoptosis inducing properties [42,43]. The antimicrobial properties of *M. oleifera* are well established. The extracts derived from *M. oleifera* tree leaves have been reported to be potential antibacterial and antifungal functions against various bacterial and fungal species [11,44,45]. Oluduro et al. [46] and Pandey et al. [47] have highlighted that *M. oleifera* exhibited 4-(α-L-rhamnopyranosyloxy) benzyl isothiocyanate, methyl N-4-(α-L-rhamnopyranosyloxy) benzyl carbamate, and 4-(α-D-glucopyranosyl-1→4-α-L-rhamnopyranosyloxy) benzyl thiocarboxamide that were able to play antimicrobial properties. The antimicrobial activities of the *Moringa oleifera* may be due to presence of lipophilic compounds and different metabolites (carboxylic acid, 2,4-diacetyl phloroglucinol, enzymes, and chitinases) in plant cell walls [48].

### 2.3. Immune Stimulating and Hypocholesterolemic Properties of M.oleifera

The immune functions of *M. oleifera* are also established by several in vitro studies [11]. Various biochemical ingredients, like quercetin, different glycosides, various isothiocyanate, kaempferol glucosides, that possess anti-inflammatory properties have been demonstrated from the extract of various parts of *M. oleifera* [49,50]. Different protein and various peptides’ (isothiocyanates, glycoside cyanides etc.) presences in *M. oleifera* leaf extracts were able to modify the immune response positively [51,52]. An investigation was carried out to detect the immunomodulatory activity of *M. oleifera* on mice model. Chronic administration of *M. oleifera* significantly increased white blood cell (WBC) count and percent of neutrophils in experimental mice [51]. The exact mechanism of action of moringa leaves on stimulating the humoral and cellular immunity is not clear yet [51]. *M. oleifera* leaf extracts are reported to possess ahypo-cholesterolemic function [53]. β-sitosterol and 4-[α-(L-rhamnosyloxy) benzyl]-o-methyl thiocarbamate (trans) are two important active substances presence in the leaf extracts of *M. oleifera* that exhibit cholesterol lowering activities. These compounds could reduce the intestine uptake of dietary cholesterol in rats [49,54]. Furthermore, plasma cholesterol was decreased and fecal cholesterol was increased in rats fed with moringa leaf extracts [49,53]. In addition, another two components, moringine and moringinine, have been recently identified from *M. oleifera* leaves, which have roles in anti-hypoglycemic functions [49,55].

### 2.4. Nutritional Properties of M.oleifera

*M. oleifera* is also very popular for its nutritional values. It is reported as a good source of six major nutrients: Carbohydrate, especially dietary fibers; proteins; vitamins; minerals; lipids; and water. The unique features of *M. oleifera* are its richness in proteins, carbohydrates, and fibers with low fat. The leaves have been reported to enclose a range of essential amino acids and are a good source of alpha linoleic acid [56]. *M. oleifera* leaves have been seen to exhibit high contents of vitamin A, C, and E [33]. The relative bioavailability of folate originated from *M. oleifera* leaves were about 82% in a rat model, which confirmed the fact that *M. oleifera* leaves exhibit rich source of dietary folate [57].

The nutritional composition of *M. oleifera* leaves (dry matter basic) showed dry matter (DM) about 93.63% to 95.0%, crude protein (CP) 17.01% to 22.23%, carbohydrate 63.11%to 69.40%, crude fiber (CF)6.77% to 21.09%, crude fat (EE) 2.11% to 6.41%, ash (total mineral) 7.96% to 8.40%, gross energy 14.790 (MJ/kg), and fatty acid 1.69% to 2.31% [58,59,60]. In addition, estimated calcium (Ca) was 1.91%; potassium (K) was 0.97%; sodium (Na) was 192.95, iron was (Fe) 107.48, manganese (Mn) was 81.65, Zinc (Zn) was 60.06, and phosphorus (P) was 30.15 parts per million (ppm) [59]. Magnesium (Mg) was 0.38%, and copper (Cu) was 6.1%, tannins 21.19%, phytates 2.57%, trypsin inhibitors 3.0%, saponins 1.60%, oxalates 0.45%, and cyanide 0.1% was also reported by Ogbe and John [59]. The leaves of the plant are enriched with methionine, phosphorus, calcium, and iron [11]. It is believed that the leaves of *M. oleifera* contain more calcium and twice as much protein than milk, higher vitamin C than oranges, higher potassium and iron than bananas, and higher vitamin A than carrots [10,61], and thus the plant is considered unique in nature [62]. Niaziridin, an active component that was identified from *M. oleifera*, can improve the absorption of different vitamins, minerals, and other micro nutrients in gastrointestinal tract of the host [50]. The nutritional composition of *M. oleifera* leaves are presented in Table 1 and Table 2.

It was thought that the moringa contains different anti-nutritional factors, such as tannins, phytates, oxalates and cyanide, which may affect normal digestion and metabolism of nutrients in animals [66]. In moringa, tannins and phytates are 12 and 21 g kg^−1^ of DM, respectively, which can be neutralized by different feed processing techniques, including chopping, socking, heat steaming, and fermentation with beneficial organisms [65]. Considering the health benefit effects of moringa, it is a unique plant due to its enriching minerals with lower anti-nutritional components [65].

## 3. Application of *M. oleifera* on Performance in Chickens

In most of the feeding experiments in poultry, the fresh, green, and undamaged mature *M. oleifera* leaves were properly air-dried, and then the dried leaves were ground to a fine powder in a hammer mill and considered as moringa leaf powder or leaf meal. Similarly, fresh mature moringa seeds were air-dried and ground and considered as moringa seed meal. In some experiments, the ground particles were then soaked into distilled water for 24 h, and the filtered aqueous solution was considered as moringa extract. Due to the rich nutrient content, especially the high amount of crude protein (CP), vitamins, and minerals, *M. oleifera* leaves can be used as a useful resource of dietary supplementation for livestock as well as poultry [65,66,67]. In addition, Briones et al. [68] stated that moringa leaves can be applied as a dietary supplement in layers and broilers due to high production performance and improved eggs quality. However, still there are many debates on the chicken’s performance with different doses of *M. oleifera* in the previous studies. There are also many variables on doses and part of plant used, such as leaves, extract, sods, or seeds. Finally, many scientists agreed that *M. oleifera* plant might have a positive role in improving the production performance and health status in chickens. Further studies are still needed to detect the actual doses of application for optimum performance in chickens.

### 3.1. Effects of M. oleifera on Growth Performance in Broilers

The major findings on the role of *Moringa oleifera* on performance in broilers are summarized in Table 3. Alabi et al. [69] applied aqueous *M. oleifera* leaf extracts on the performance in broiler chickens. This study demonstrates that average daily body weight gain and final body weight were higher in 120 mL/L extract-supplemented groups than the control. Feed intake was highest in birds on positive control (having antibiotics) and lowest in birds that consumed 90 mL/liter of leaf extracts. Feed conversion ratio (FCR) was lower in birds on 90 mL/L and 120 mL/L of leaf extracts fed groups. Collectively, the authors suggested that moringa leaf extracts can be added up to 90 mL/L in broiler chickens for optimum performance. The higher body weight and lower FCR in this study might be related to the presence of different bioactive components in moringa leaf extracts that may play a role in improved nutrient utilization in supplemented birds. Similarly, higher body weight was also recorded by Khan et al. [70] who used moringa leaf powder as dietary supplement with 1.2% levels in broilers. Abdulsalam et al. [71] conducted an experiment with moringa leaf meal in broilers and found that supplemented diets could enhance the growth performance at finisher period. The authors finally stated that moringa leaf meal can be applied as a natural source of protein in broiler diets. Similarly, inclusion of *Moringa oleifera* leaves at higher levels (15% and 20%) in broiler diets resulted in a higher growth rate and better health status in broilers [14]. In addition, dietary supplementation of *M. oleifera* leaves at 5% to 20% level showed higher growth performance in broilers [66]. Final live weight, average weight gain, and FCR were higher in 10% moringa leaf meal supplemented diets than the control through a 35-day trial period [72].Furthermore, feeding with *M. oleifera* leaf powder could improve live weight, body weight gain, dressing percentage, and FCR in broilers [73].

In contrast, no significant differences were observed on growth performance and economic parameters in broilers fed with *Moringa oleifera* leaf meal, according to Onunkwo and George [18]. Finally, the authors stated that *Moringa oleifera* leaf meal may be used at the level of 10% with view to reducing the production cost [18]. Similarly, feeding with moringa leaf meal in broilers led to a lower feed intake with higher FCR, as reported by Gakuyaet al. [74], which was due to presence of anti-nutritional factors in moringa leaves used in the experiment diets as row basis. No significant differences were observed on final live weight and dressing percentage by feeding moringa seed powder in broilers [75]. Gadzirayi et al. [76] applied *Moringa oleifera* leaf meal as supplementing part of conventional soybean meal in broiler diets at 0%, 25%, 50%, 75%, and 100% level. The author did not find any significant differences on feed intake and body weight gain between control and 25% level of moringa supplementation. However, significantly lower FCR was observed in moringa leaf meal fed groups. Finally, the study suggested using moringa leaf meal at a 25% level to promote growth in broilers. In addition, Ayssiwede et al. [77] noted that dietary application of moringa leaf meal up to a level of 24% had no adverse effects on body weight, average daily weight gain, FCR, mortality, and the weight of organs in broilers compared to the control diet. Olugbemi et al. [78] stated that average daily growth rate was lower with *Moringa oleifera* leaf meal at the inclusion level below 5% in diets, and the authors suggested to use maximum level of 5% without any harmful effects on growth performance and FCR in broilers. These findings confirmed the fact that feeding with moringa leaves had no deleterious effects on normal physiology and growth in the experimental broilers. However, collectively, some authors suggested that use of the *Moringa oleifera* leaf meal up to a 10% level would not have any adverse effects in broilers [78,79,80]

### 3.2. Effects of M. oleifera on Meat and Bone Quality in Broilers

Dietary manipulation is an important way to improve the meat quality in poultry [2]. The meat derived from broiler chickens is an excellent source of protein, vitamins, minerals, and lower fat and has created a great demand among consumers [88]. Meat pH, tenderness, color (lightness, redness, and yellowness),and water holding capacity are very important meat quality characteristics to the consumers. An experiment on supplementation of *Moringa oleifera* leaf powder on the quality of meat and bone in broilers was conducted by Rehman et al. [81]. This study noticed that supplementation of leaf powder at 12 g/kg level could increase pH, water holding capacity, and muscle fiber diameter in the breast muscle of experimental broilers. In addition, higher weight, ash percentage, and the density of tibia bone in broilers fed with moringa leaf meal were also recorded in their studies [81]. In this study, authors hypothesized that higher muscle pH values in experimental groups were due to the stabilization of the myofibrils by activating antioxidant properties and preventing free radicals. Higher breast muscle weight could be the result of increased protein deposition in moringa-supplemented groups. The higher tibia bone weight and ash percent may be due to the presence of phytoestrogen flavonoids in moringa leaves powder. In contrast, Nkukwana et al. [82] found that *Moringa oleifera* leaf meal had no effects on tibia bone characteristics but could improve body weight gain and FCR. These differences might be related with inclusion levels and types of incorporation of moringa in broiler diets. However, it is a popular belief that dietary antioxidants can modify the meat color, minimize the rancidity, and retard lipid peroxidation, resulting in a well-maintained meat quality. The oxidative status of meat muscle is directly related to meat quality and has negative effects on cooking loss, drip loss, meat color, and pH [89]. Therefore, dietary supplementation of antioxidant-enriched moringa leaves would be a potential strategy to improve the meat quality in broilers. Moreover, it was reported that phytosterols could reduce malondialdehyde (MDA) content and increase glutathione (GSH) concentration in the breast muscle of experimental broiler chickens [88]. The inclusion of moringa leaf meal could improve fatty acid profile and could reduce lipid oxidation in breast muscle of broilers [84]. The authors assumed that improved fatty acid profile was due to the presences of saturated fatty acids in moringa leaves. 

### 3.3. Effects of M. oleifera on Health Status in Broilers

Alnidawi et al. [14] has conducted an experiment with a view to examining the effects of *Moringa oleifera* leaf on health status in broilers.This study ensured that total cholesterol content was lower with higher level (at 15% and 20%)of *M. oleifera* fed in broiler diets. Similarly, high-density lipoprotein cholesterol (HDL) content in serum was increased and low-density lipoprotein cholesterol (LDL) was decreased with higher level of supplementation of *M. oleifera* in broilers. It was hypothesized that higher amounts of natural fiber in moringa leaves may have a role in lowering cholesterol level by increasing lipid metabolism in the host body. In addition, the blood parameters, like hemoglobin percent, total red blood cells number, and total packed cell volume, were found to be higher at 20% supplementation levels than the control diet [14]. *M. oleifera* leaf powder was considered as dietary supplement with 0.6%, 0.9%, 1.2% and 1.5% levels in broilers on growth performance and intestinal microarchitecture [70]. The intestine’s morphological characteristics in chickens are vital for nutrient utilization and an indicator of sound physiology. The length and empty weight of small intestine were found higher in broilers fed with 1.2% leaves powder. In addition, higher villus height (duodenum, jejunum, ileum), villus surface area (duodenum), and villus height/crypt depth (ileum) were observed in 1.2% leaves powder fed group than the control. Higher villi suggest better absorption of nutrients due to enlarged surface area, which is a good indicator of gut system. Furthermore, the improvement of villus height and villus height/crypt depth ratio may be linked with high content of crude fiber in moringa-supplemented diets. This study further observed that total goblet cells of duodenum were higher in broilers fed with all levels of *M. oleifera* leaf powder in comparison with control group. The findings indicate enhanced mucosal protection with *M. oleifera* supplementation in broiler diets. Goblet cells are essential elements of innate gut immune system in poultry. Bursal follicle count was also found to be higher in 1.2% *M. oleifera*-fed group than non-supplemented control diet. Finally, the authors concluded that dietary supplementation of *M. oleifera* at 1.2% level could modulate the intestinal structure and acidic mucin production without any adverse effects on growth performance in broilers [70]. 

The extract from the leaves of *Moringa oleifera* has apotential role as an anti-bacterial and antioxidant functions [22]. The roles of *Moringa oleifera* leaf meal at 10% and 15% level on the hematological parameters in broilers were examined by Ebenebe et al. [72]. Feeding *Moringa* leaf meal in broilers resulted in increased red blood cell (RBC), packed cell volume (PCV), and hemoglobin (HB) values in both levels of diets. Finally, the authors stated that *Moringa oleifera* leaf meal should be used within the 10% level in broiler diets. *Moringa oleifera* is known to a potential antioxidant with some antioxidant properties due to the presence of vitamins C and E, carotenoids, flavonoids, and selenium [15]. *Moringa oleifera* leaves contain various phytochemicals (carotenoids, flavonoids, chlorophyll, phenolics, xanthins, cytokines, alkaloids, etc.) that might have a role in improving health status [90]. 

### 3.4. Effects of M. oleifera on Egg Production, Performance, and Egg Quality in Laying Hens 

The major findings on the role of *M. oleifera* in performance in laying hens are summarized in Table 4. The egg quality parameters, including egg size, shape, color, shell thickness, and egg yolk cholesterol, directly and indirectly influence egg consumers. In a recent study by Voemesse et al. [91], *M. oleifera* leaf meal was used in layer chickens’ diet from 1 day old to 55 weeks of age to investigate the effects of moringa leaf meal on growth performance, egg production performance, and blood parameters. *M. oleifera* leaf meal was used at three different levels (0%, 1%, and 3%). In the growing period from 1 day to 20 weeks of age, this study did not find any significant differences on feed intake, but average daily body weight gain, final body weight, and FCR were improved in *M. oleifera*-supplemented groups. In the laying period, from 21 weeks to 55 weeks, feed intake was lower in moringafed groups, but the laying percent and FCR were higher in supplemented fed groups than the non-supplemented group. The higher body weight gain and egg production may be related to improved digestibility in supplemented groups due to different active components in moringa leaves. The author concluded that feeding moringa leaf meal at 1% level had positive effects on the growth and egg production in laying hens.In addition, *Moring oleifera* at 10% levels showed higher egg production in laying hens [66]. According to Abouz-Elezz et al. [92], *M. oleifera* supplementation could improve the egg production, egg mass, and egg yolk color scores compared with the non-supplemented groups. The improvement of yolk color scores could be due to high carotene content in moringa leaves. Higher feed intake, crude protein intake, weight gain, FCR, and protein efficiency ratios were recorded in laying chicks where *Moringa stenopetala* was the experimental supplement [93]. This is because of readily available proteins with their essential amino acids in the moringa leaf meal. The authors finally concluded that *Moringa stenopetala* leaf meal at up to 6% levels can be applied in growing chicks’ ration.

In contrast, *Moringa oleifera* seed meal at 0%, 1%, 3% and 5% levels were used to examine the effects of egg production performance, egg quality, and egg fatty acid profile in Hy-Line laying hens [94]. Lower feed intake, egg production percent, egg mass, feed intake, and body weight were observed in moringa seed meal-fed groups than the control. Higher egg yolk color scores with higher linolelaidic acid in egg yolk were found in moringa seed meal supplemented groups than the non-supplemented diets [94]. The moringa seeds may contain different anti-nutritional factors, which may have deleterious effects on production performance in this study. In addition, Ahmad et al. [95] also reported that the decrease in production performance of layer chickens was due to high fiber and different anti-nutritional factors’ presences in moringa pod meal. However, this study found a significant positive role in improving β-carotene, quercetin, and selenium levels in egg yolk with moringa pod supplementation. Moringa pods are naturally enriched with carotenoids and different flavonoids, which possess natural antioxidants that could modify the β-carotene and quercetin levels in egg yolk [74]. Egg yolk cholesterol was significantly lower in moringa pod meal fed groups than the control group, which may be due to presence of natural antioxidants in the experimental diets containing moringa pod meal in this study. In addition, the nutrient profile of egg yolk was higher with the supplementation of moringa pod meal in Hy-Line layers [95]. In another study, Lu et al. [96] found that *M. oleifera* leaf meal had no effects on egg production, egg weight, and feed intake in Hy-Line Grey commercial layers, but birds fed with moringa leaf meal at 15% levels showed deeper egg yolk color than the non-supplemented fed group. Similarly, the albumen height and Haugh unit were higher in moringa-supplemented groups during storage of eggs at 4 °C and 28 °C for 4 weeks. Finally, the author stated that 5% moringa leaves meal can be included in laying hens’ ration without adverse effects on egg production and egg quality. Similarly, Abou-Elezz et al. [80] found that *Moringa oleifera* leaf meal could improve egg yolk color scores and albumen percentage. This study further observed the lower egg laying percentage and egg mass in laying hens fed with moringa leaf meal. However, this study did not find any significant differences on final body weight and on other egg quality parameters (yolk percent, shell percent, and shell thickness). Finally, the author stated that 10% moringa leaf meal can be incorporated into the diets of Rhode Island Red laying hens. Feed intake, feed conversion ratio, and laying percentage were not influenced by adding moringa leaf meal at a 10% level, which was noticed by Olugbemi et al. [78]. However, inclusion of 10% moringa leaf meal could increase higher egg Roche color score [78]. A similar report on decreased egg mass and egg production percent with moringa leaf meal supplementation at higher levels (at 10% and 20%) in laying hens was observed by Kakengi et al. [79]. Interestingly, *Moringa oleifera* leaf meal at 5% level increased the egg weight, but the decreased egg weight was found when inclusion level was at 20%. The authors assumed that higher feed intake, FCR with lower egg production percent, egg mass, and egg weight at a higher-level supplementation was due to poor digestibility of nutrients because of different anti-nutritional phytochemical presences in moringa leaves [79].

Improving the egg quality by means of increasing its anti-oxidative properties by supplementing natural unconventional resources has gained a significant interest in poultry research [97]. The synthesis antioxidants, like butylated hydroxyanisole and butylated hydroxytoluene, are commonly used in food processing. However, they are found to be carcinogenic to human health, therefore, discovering natural antioxidant products as safe and effective alternatives is a very crucial need [98,99,100].

### 3.5. Effects of M. oleifera on Health Status in Laying Hens

Analyzing blood parameters is very important in detecting the health status of birds. According to Voemesse et al. [91], serum albumin level was higher in laying hens fed with 3% level of moringa leaf meal than the control group, but the number of white blood cells (WBCs),red blood cells (RBCs), lymphocytes, and the packed cell volume were lower in moringa-fed groups than the control diets. The authors assumed that lower WBCs and lymphocytes in moringa-fed chickens may be due to the antimicrobial activity of phytochemicals in the moringa leaves. It is well known that a high WBC count is related to an infection caused by bacteria in the host. Lower level of cholesterol content in serum with dietary supplementation of moringa pod meal were observed, which might be influenced by antioxidants (flavonoids and carotenoids) and high fiber presences in the moringa pod meal in the experimental diets [95]. However, this study did not find any significant differences on antibody response against Newcastle disease virus. Lower values for malondialdehyde (MDA) and higher glutathione peroxidase in the plasma of laying hens fed with moringa leaf meal indicated the higher antioxidant activities [96]. Plasma total protein levels were higher by dietary 5% for moringa leaf meal supplementation, which is a good indicator of the liver’s synthetic function. Furthermore, lower plasma uric acid in supplemented groups indicated higher protein retention in laying hens [96]. The improved antioxidant enzyme activities and the reduced MDA levels in the plasma and egg yolk indicated the fact that dietary moringa supplementation could improve the antioxidant activities. *Moringa oleifera* is an effective phytobiotic and is known to possess broad-spectrum antibacterial properties and immuno-modulatory functions [70,81,102]. 

## 4. Conclusions 

This review study highlights that *M. oleifera* could be fruitfully used as an effective natural growth promoter as well as an immune-boosting agent in chickens’ ration. Although *M. oleifera* was used in the experimental diets of poultry, further study was recommended by various researchers regarding the doses of *M. oleifera* on optimum performance and sound health in chickens. Thus, the future study should beconducted ina proper way so that it will examine the uses of *M. oleifera* in reaction to a pathogen challenge as well as dosages. In this study, we suggest future research with *M. oleifera* as an alternative for antibiotics in chickens so that it may be used as an effective strategy for organic meat and egg production. It could be concluded that *M. oleifera* can be used as an environmentally friendly feed supplement in chicken ration. The present study will help future researchers to discover the important effects of *M. oleifera* on immunity and health status that the past studies were not able to explore. Thus, the supplementation with *M. oleifera* may be a new concept of research in chicken production. The inclusion level of *M. oleifera* up to 10% in both broilers’ and laying hens’ diet could be recommended.

## Figures and Tables

**Table 1:** Chemical compositions of *Moringa oleifera* leaves ^†^.

Nutrient Component	Fresh Leaves	Dry Leaves	Leaves Powder
Calories (cal)	92	329	205
Protein (g)	6.7–17.1	29.4–40.0	25.4–27.1
Fat (g)	1.7–2.11	5.2–6.5	2.3
Carbohydrate (g)	6.3–12.5	38.0–41.2	34.3–38.2
Fiber (g)	0.9–7.09	12.5–21.09	19.2
Vitamin A	0.9–11.05	16.3–18.90	-
Vitamin B1 (mg)	0.06	2.02–2.60	2.64
Vitamin B2 (mg)	0.05	19.82–21.3	20.5
Vitamin B3 (mg)	0.8	7.6–8.3	8.2
Vitamin C (mg)	220	15.8–17.3	17.3
Vitamin E (mg)	448	10.8–77.0	113
Calcium (mg)	440	2185–3050	2003
Magnesium (mg)	42–82	86–448	368
Phosphorus (mg)	30.15–70	204–252	204
Potassium (mg)	259	1236–1384	1324
Copper (mg)	0.07	0.08–0.49	0.57
Iron (mg)	0.85–10.7	25.6–490	28.2
Sulphur (mg)	-	363–630	870
Zinc (mg)	6.7	3.25–13.03	-
Manganese (mg)	81.6	86.8–91.2	-

^†^ All values are in 100 g per plant material. References: [10,56,63].

**Table 2:** Amino acid contents in *Moringa oleifera* leaves ^†^.

Amino Acid	Fresh Leaves (mg g^−1^ DM)	Extracted Leaves (mg g^−1^ DM)
Lysine	13.25–26.77	14.06–18.09
Leucine	20.52–42.89	17.5–21.84
Isoleucine	11.91–22.53	8.08–11.30
Methionine	3.5–8.96	1.13–4.97
Cystine	3.8–5.18	1.0–3.39
Phenylalanine	16.31–27.14	8.9–15.51
Tyrosine	18.88	9.71
Valine	10.62–27.58	7.25–14.26
Histidine	5.17–13.57	7.16–7.50
Threonine	13.5–21.97	7.90–11.70
Serine	10.87–20.79	9.40–10.34
Glutamic acid	28.42–50.85	17.10–25.65
Aspartic acid	20.52–46.11	14.3–22.16
Proline	14.3–25.75	12.41–13.63
Glycine	15.33–26.62	10.3–13.73
Alanine	28.67–30.33	12.51–18.37
Arginine	18.9–30.28	13.25–15.64
Tryptophan	4.25–9.26	5.27–7.16

^†^ References: [15,56,64,65].

**Table 3:** Role of *Moringa oleifera* on performance in broilers. ^†^

Types	Study Design	Main Findings	References
*Moringa oleifera* leaf powder	broilers (Hubbard) from 1–35 days, dose: 6,9,12, and 15 g/kg (supplementation type)	●higher pH of breast muscle ●higher weight and diameter of breast muscle fibers ●higher water holding capacity of breast muscle ●higher weight length index of tibia bone ●higher ash percentage of tibia bone ●no effects on alkaline phosphatase in tibia bone	[81]
*Moringa oleifera* leaf powder	broilers (Hubbard) from 1–35 days, dose: 0, 0.6%, 0.9%, 0.12%, 0.15% (supplementation type)	●no effects on feed intake, FCR and bursa weight ●higher final body weight ●higher length of small intestine ●higher empty weight of small intestine and ceca ●higher villus height (duodenum, jejunum, ileum) ●higher villus height/crypt depth (ileum) ●higher goblet cell number (total) in duodenum ●higher acidic mucin number in duodenum, jejunum and ileum	[70]
*Moringa oleifera* leaf extract	broilers (Hubbard)from 1–42 days, dose: 0, 60, 90, 120, 150 mL/L	●higher body weight gain ●lower FCR ●no effects on weight of inner organs ●no effects on dressing percentage	[69]
*Moringa oleifera* leaf meal	broilers from 0–42 days, dose: 0, 5%, 10% 15%, 20%, (inclusion type)	●higher body weight ●higher hemoglobin percent, and RBC number ●lower TC, LDL	[14]
*Moringa oleifera* seed powder	broilers from 1–42 days, dose: 0, 0.5%, 0.1%, and 2% (inclusion type)	●no effects on live weight and weight gain ●no effects on FCR ●no effects on dressing percentage, liver weight and heart weight	[75]
*Moringa oleifera* leaf meal	broilers (ANIK 2000 strain) from 0–49 days, dose: 0, 5%, 7.5%, 10 % (inclusion type)	●higher dressing weight in 7.5% and 10% level ●higher weight of liver, spleen, and gizzard ●no significant effects on body weight gain, feed intake and FCR	[18]
*Moringa oleifera* leaf meal	broilers (Cobb-500) from 1–35 days, dose: starter (1, 3 and 5 g/kg); grower (3, 9, and 15 g/kg); and finisher (5, 15, and 25 g/kg) (inclusion type)	●higher body weight and weight gain at grower period ●lower FCR ●no effects on feed intake ●higher Ca and P content in tibia bone ●no effects on tibia weight, tibia length, and weight-length index of tibia bone ●no effects on ash content in tibia, and bone breaking strength	[82]
*Moringa oleifera* leaf meal	broilers (Cobb-500) from 1–35 days, dose: starter (1, 3, and 5 g/kg), grower (3, 9, and 15 g/kg) and finisher (5, 15, and 25 g/kg) (inclusion type)	●higher body weight at starter and finisher period ●lower FCR ●no effects on feed intake ●higher dressing percentage, thigh muscle weight and bursa weight ●no effects on CP, CF, DM, EE, ash, NDF, ADF digestibility	[83]
*Moringa oleifera* leaf meal	broilers (Cobb-500) from 1–35 days, dose: 1%, 3%, and 5% (inclusion type)	●higher body weight and weight gain at starter period ●lower FCR ●no effects on feed intake ●higher thiobarbituric acid reactive values in breast muscle during storage ●higher fatty acid profile (C18:0, C15:0, C20:0, C20:3n6 and C22:6n3) levels ●no effects on thrombogenic index and atherogenic index in breast muscle	[84]
*Moringa oleifera* leaf meal	broilers (Ross) from 1–49 days, dose: 0, 3%, 5%, and 7% (inclusion type)	●higher final body weight and weight gain ●lower FCR ●higher feed intake ●higher dressing percentage ●higher meat tenderness and juiciness score	[85]
*Moringa oleifera* leaf meal	broilers from 1–42 days, dose: 0, 7.5%, 15%, and 30% (inclusion type)	●lower final body weight and weight gain ●higher FCR ●lower dry matter digestibility ●no effects on crude protein, crude fiber digestibility ●no effects on lipid metabolic profile (HDL, TC, LDL) ●higher meat color scores	[74]
*Moringa oleifera* leaf meal	broilers from 1–35 days, dose: 0, 10%, 15% (inclusion type)	●higher body weight gain ●lower FCR ●higher final body weight ●higher RBC number, PCV number, and HB percent	[72]
*Moringa oleifera* leaf meal	broilers (Habbard) from 0–42 days, dose: 0, 25%, 50%, 75 %, 100% (supplementation type)	●no effects on feed intake ●no effects on weight gain ●lower FCR	[76]
*Moringa oleifera* leaf powder	broilers from 1–42 days, dose: 0, 0.05%, 0.10% (supplementation type)	●higher body weight gain ●lower FCR ●higher final body weight ●higher dressing percentage	[73]
*Moringa oleifera* leaf extract	broilers (Cobb)from 1–35 days, dose: 0, 30, 60, 90 mL/L	●higher live weight ●lower FCR ●higher returns to investment ●lower feed intake	[86]
*Moringa oleifera* leaf meal	broilers (Cobb) from 14–42 days, dose: 0, 5%, 10%, and 15% (inclusion type)	●lower weight gain and final body weight ●higher FCR ●no effects on dressing percentage and carcass weight ●no effects on weight of inner organs ●no effects on CP and EE content in meat ●no effects on total cholesterol, HDL, LDL, total protein, glucose	[87]

^†^ FCR, feed conversion ratio; HDL, high density lipoprotein cholesterol; TC, total cholesterol; LDL, low density lipoprotein cholesterol; RBC, red blood cell; PCV, packed cell volume; HB, hemoglobin; CP, crude protein; CF, crude fiber; DM, dry matter; EE, ether extract; NDF, neutral detergent fiber; ADF, acid detergent fiber.

**Table 4:** Role of *Moringa oleifera* on performance in laying hens.^†.^

Types	Study Design	Main Finding	References
*Moringa oleifera* leaf meal	laying chickens and laying hens, from 1 day to 55 weeks, dose: 0, 1%, 2%, and 3% (inclusion type)	●no effects on feed intake in growing period ●higher body weight gain, and lower FCR ●lower feed intake in laying period ●higher egg production ●lower FCR ●higher serum albumen percent ●lower number of RBCs, WBCs, PVC, and lymphocytes	[91]
*Moringa oleifera* seed meal	laying chickens (Hy-Line), from 20–28 weeks, dose: 0, 1%, 3%, and 5% (inclusion type)	●lower egg production percent, egg mass, feed intake and body weight ●no effects on egg weight ●lower egg albumen height ●higher egg yolk color scores ●no effects on albumen weight, yolk weight, egg shell weight, and egg shape index ●lower palmitoleic acid in egg yolk ●higher linolelaidic acid in egg yolk	[94]
*Moringa oleifera* pod meal	laying hens (Hy-Line), from 50–66 weeks, dose: 0, 5, 10 and 15 g/kg (inclusion type)	●higher egg mass and lower FCR ●no effects on egg production, egg weight, and feed intake ●higher Haugh unit and egg shell thickness ●no effects on egg shape index and egg yolk index ●higher β-carotene, quercetin, and selenium in egg yolk ●lower cholesterol in egg yolk and serum ●higher nutrient composition in egg yolk	[95]
*Moringa oleifera* leaf meal	laying hens (Hy-line Gray) from 27–35 weeks, dose: 0, 5%, 10%, and 15% (inclusion type)	●no effects on feed intake and egg weight ●higher FCR ●lower egg production ●higher egg yolk color scores ●higher albumen height and Haugh unit during storage ●higher glutathione peroxidase in plasma ●lower malondialdehyde and uric acid in plasma	[96]
*Moringa oleifera* leaf powder	laying hens (Lohmann LSL) from 27–40 weeks, dose: 0, 0.2%, 0.4%, 0.6%, 0.8% (inclusion type)	●no effects on egg production, egg weight, and FCR ●no effects on sensory evaluation of eggs quality ●no effects on egg shell thickness	[101]
*Moringa oleifera* leaf meal	laying hens (Rhode Island Red, RIR) from 27–38 weeks, dose: 0, 300 g/kg, (supplementation type)	●higher egg production ●higher egg mass ●higher egg yolk color scores ●lower FCR ●higher albumen percent	[92]
*Moringa oleifera* leaf meal	laying hens (Rhode Island Red, RIR) from 36–41 weeks, dose: 0, 5%, 10%, and 15% (inclusion type)	●no effects on final body weight, egg weight, and FCR ●decreased egg production ●decreased egg mass ●higher egg yolk color scores ●higher albumen percent ●no effects on yolk percent, shell percent, and shell thickness	[80]
*Moringa oleifera* leaf meal	laying hens from 65–73 weeks, dose: 0, 5%, 10%, and 20% (inclusion type)	●no effects on feed intake, egg production percent, and FCR ●decreased feed cost ●no effects on albumen and yolk percent ●higher egg Roche color scores	[78]

^†^ FCR, feed conversion ratio; RBC, red blood cell; WBC, white blood cell; PCV, packed cell volume.
